# Diagnosing Coronary Artery Disease via Data Mining Algorithms by Considering Laboratory and Echocardiography Features

**DOI:** 10.5812/cardiovascmed.10888

**Published:** 2013-07-31

**Authors:** Roohallah Alizadehsani, Jafar Habibi, Zahra Alizadeh Sani, Hoda Mashayekhi, Reihane Boghrati, Asma Ghandeharioun, Fahime Khozeimeh, Fariba Alizadeh-Sani

**Affiliations:** 1 Department of Computer Engineering, Sharif University of Technology, Tehran, IR Iran; 2 Rajaie Cardiovascular Medical and Research Center, Tehran University of Medical Science, Tehran, IR Iran; 3 Mashhad University of Medical Sciences, Mashhad, IR Iran

**Keywords:** Data Mining, Sensitivity and Specificity, Coronary Artery Disease

## Abstract

**Background:**

Coronary artery disease (CAD) is the result of the accumulation of athermanous plaques within the walls of coronary arteries, which supply the myocardium with oxygen and nutrients. CAD leads to heart attacks or strokes and is, thus, one of the most important causes of death worldwide. Angiography, an imaging modality for blood vessels, is currently the most accurate method of diagnosing artery stenosis. However, the disadvantages of this method such as complications, costs, and possible side effects have prompted researchers to investigate alternative solutions.

**Objectives:**

The current study aimed to use data analysis, a non-invasive and less costly method, and various data mining algorithms to predict the stenosis of arteries. Among many people who refer to hospitals due to chest pain, a great number of them are normal and as such do not need angiography. The objective of this study was to predict patients who are most probably normal using features with the highest correlations with CAD with a view to obviate angiography costs and complications. Not a substitute for angiography, this method would select high-risk cases that definitely need angiography.

**Patients and Methods:**

Different features were measured and collected from potential patients in order to construct a dataset, which was later utilized for model extraction. Most of the proposed methods in the literature have not considered the stenosis of each artery separately, whereas the present study employed laboratory and echocardiographic data to diagnose the stenosis of each artery separately. The data were gathered from 303 random visitors to Rajaie Cardiovascular, Medical and Research Center. Electrocardiographic (ECG) data were studied in our previous works. The goal of this study was, therefore, to seek the accuracy of echocardiographic and laboratory features to predict CAD patients that require angiography.

**Results:**

Bagging and C4.5 classification algorithms were drawn upon to analyse the data, the former reaching accuracy rates of 79.54%, 61.46%, and 68.96% for the diagnosis of the stenoses of the left anterior descending (LAD), left circumflex (LCX), and right coronary artery (RCA), respectively. The accuracy to predict the LAD stenosis was attained via feature selection. In the current study, features effective in the stenosis of arteries were further determined, and some rules for the evaluation of triglyceride, hemoglobin, hypertension, dyslipidemia, diabetes mellitus, and ejection fraction were extracted.

**Conclusions:**

The current study presents the highest accuracy value to diagnose the LAD stenosis in the literature.

## 1. Background

Data mining is the process of exploring hidden knowledge in a database. This science extracts patterns by combining statistics and artificial intelligence methods as well as database management techniques. Nowadays, data mining usage has spread in a large number of fields, including marketing, fraud detection, and knowledge discovery.(1). Mortality rates of diseases are much higher than those of accidents and disasters. The major culprit for death in most developing countries such as India and China is cardiovascular disease(2). Cardiovascular disease is a heart and blood vessel disease, creating numerous problems, many of which are related to a process called atherosclerosis. Atherosclerosis is a condition that emerges when a substance called plaque accumulates in the walls of arteries, thereby obstructing the blood flow through vessels and probably leading to a heart attack or stroke. Angiography is the traditional gold standard to evaluate vascular lesions. However, this diagnostic modality is complicated, costly, and can have side effects.

Data mining is utilized as an alternative detection method for the prediction of coronary artery disease (CAD) with high accuracy, based on a set of features collected from the patient. Most of the studies conducted thus far have not considered the stenosis of each artery separately. In such methods, if each of the left anterior descending (LAD), left circumflex (LCX), and right coronary artery (RCA) is stenotic, the existence of CAD is concluded. A few studies have considered the stenosis of each artery separately so far. Babaoglu et al. (3) used exercise test data and neural networks to study the stenosis of each artery. They reached accuracy rates of 73%, 64.85%, and 69.39% to diagnose the stenosis of the LAD, LCX, and RCA, respectively. Alizadehsani et al. (4) ran the Naïve Bayes and KNN algorithms on symptom and examination features as well as the electrocardiographic (ECG) features and attained accuracy rates of 74.20%, 63.76%, and 68.33% to diagnose the stenosis of the LAD, LCX, and RCA, respectively. The difference between these two studies is because of the different features selected in these two papers. Srinivas et al. (5) and Ordonez et al. (6) extracted some rules for the stenosis of each artery. Sony et al. (7) found a number of rules for the stenosis of each vessel, by means of decision tree.

In addition to diagnosing the stenosis of arteries, some articles have studied the presence of CAD in individuals. Alizadehsani et al. (8)examined the SMO, Naïve Bayes algorithms, and a new ensemble method on symptom and examination features in conjunction with the ECG features and obtained an accuracy rate of 88.5% to diagnose CAD. The C4.5 and Bagging (which consist of C4.5) algorithms were used in the present study in order to diagnose the stenosis of the LAD, LCX, and RCA arteries.

## 2. Objectives

This study aimed to draw upon data mining algorithms to diagnose the stenosis of arteries and find some rules based on the dataset features. The main goal of this study was to achieve a reliable estimate of presence of significant CAD in patients presenting with chest pain based on a series of readily accessible data, including history, cardiac risk factors, and echocardiographic and laboratory tests, with a view to reducing the number of patients undergoing angiography and, thus, decreasing hospital admission durations and financial burdens on health care systems.

## 3. Patients and Methods

The dataset of the current study was gathered from 303 random individuals, who referred to Rajaie Cardiovascular, Medical and Research Center, Tehran, Iran, due to chest pain.

### 3.1. C 4.5

The C4.5 is a classification algorithm based on decision trees and was presented to augment the performance of simple decision trees. The difference between ID3, which is one of the primitive decision-tree algorithms, and C4.5 is the ability of the latter to manage continuous values by breaking them down into sub intervals. Also, the C4.5 uses pruning methods to improve accuracy and avoid memorizing the test data (9). In the current study, confidence threshold for pruning and the minimum number of instances per leaf were set at 0.25 and 2, respectively.

The data mining algorithms used in the current study are described in this section. The default settings, as defined by RapidMiner software, were employed in both C4.5 and Bagging algorithms. The accuracy of these two algorithms was calculated using ten-fold cross-validation method.

### 3.2. Bagging

The Bagging classifies each sample based on the output of a set of diverse base classifiers. The base classifiers can be selected from the C4.5, Naïve Bayes, ID3, and other data mining algorithms (10). In the present study, the C4.5 was used and the number of weak learners was set at 10.

## 4. Results

Descriptive findings about the patients’ demographic and background data are presented in tables 1 and 2. (version of 5.2) tool was employed. RapidMiner is a tool to process machine learning and data mining algorithms (11). The present study made use of the default setting of the RapidMiner and utilized accuracy, sensitivity, and specificity to perform the algorithms. These quantities are described in what follows.

**Table 1.** Demographic Features

Demographic Features	Descriptive Index	Range
**Age, year**	58.898 ± 10.392	**(30-86)**
**Weight,Kg**	73.832 ± 11.987	**(48-120)**
**Sex**		
Male	176 (58.1%)	
Female	127 (41.9%)	
**BMI^a^, Kg/m^2^**	27.248±4.099	**(18-41)**
**DM^a^**		
Yes	90 (29.7%)	
No	213 (70.3%)	
**HTN^a^**		
Yes	179 (59.1%)	
No	124 (40.9%)	
**Current Smoker**		
Yes	63 (20.8%)	
No	240 (79.2%)	
**Ex-Smoker**		
Yes	10 (3.3%)	
No	293 (96.7%)	
**FH^a^**		
Yes	48 (15.8%)	
No	255 (84.2%)	
**Obesity**		
Yes if BMI > 25	211 (69.6%)	
No, otherwise	92 (30.4%)	
**CRF^a^**		
Yes	6 (2%)	
No	297 (98%)	
**CVA^a^**		
Yes	5 (1.7%)	
No	298 (98.3%)	
**Airway Disease**		
Yes	11 (3.6%)	
No	292 (96.4%)	
**Thyroid Disease**		
Yes	7 (2.3%)	
No	296 (97.7%)	
**CHF^a^**		
Yes	1 (0.3%)	
No	302 (99.7%)	
**DLP^a^**		
Yes	191 (63%)	
No	112 (37%)	

^a^ Abbreviations: BMI, body mass index; DM, diabetes mellitus; HTN, hyper tension; FH, family history; CRF, chronic renal failure; CVA, cerebrovascular accident; CHF, congestive heart Failure; DLP, dyslipidemia

**Table 2.** Laboratory and Echo Features

Laboratory Features^a^	Descriptive Index	Range
**FBS^b^**	119.185 ± 52.08	62-400
**Cr^b^**	1.056 ± 0.264	0.5-2.2
**TG^b^**	150.343 ± 97.959	37-1050
**LDL^b^**	104.644 ± 35.397	18-232
**HDL^b^**	40.234 ± 10.559	15-111
**BUN^b^**	17.502 ± 6.957	6-52
**ESR^b^**	19.462 ± 15.936	1-90
**HB^b^**	13.153 ± 1.61	8.9-17.6
**K^b^**	4.231 ± 0.458	3.0-6.6
**Na^b^**	140.997 ± 3.808	128-156
**WBC^b^**	7562.046 ± 2413.739	3700-18000
**Lymph^b^**	32.399 ± 9.973	7-60
**Neut^b^**	60.149 ± 10.182	32-89
**PLT^b^**	221.488 ±60.796	25-742
**EF^b^(%)**	50.788 ± 8.652	15-60
**Region with RWMA^b^**	0.620 ± 1.133	
**VHD^b^**		
Normal	116 (38.3%)	
Mild	149 (49.2%)	
Moderate	27 (8.9%)	
Severe	11 (3.6%)	

^a^ Valvular diseases in the current study were classified based on their severity and not on the basis of which valve was diseased. Furthermore, patients with known or predominant valvular heart disease were excluded from the study.

^b^ FBS, fasting blood sugar; C, Creatine; TG, triglyceride; LDL, low-density lipoprotein; HDL, high-density lipoprotein; BUN, blood urea nitrogen; ESR, erythrocyte sedimentation rate; HB, hemoglobin; K, potassium; Na, sodium; WBC, white blood cell; Lymph, lymphocyte; Neut, neutrophil; PLT, platelet; EF, ejection fraction; RWMA, regional wall motion abnormality; VHD, Valvular Heart Disease

### 4.1. Confusion Matrix

Performance quantities were measured by means of the confusion matrix. Table 3 depicts a general confusion matrix, in which positive means having the disease and negative means being healthy.

**Table 3.** Confusion Matrix

	Actual Positive	Actual Negative
**Predicted Positive**	a	c
**Predicted Negative**	b	d

### 4.2. Performance Measures

Sensitivity, specificity, and accuracy are described below based on the confusion matrix(12).

sensitivity = *α* / *α + b*

specificity = *d* / *d + c*

accuracy = *(a+d)* / *(a+b+c+d)*

Sensitivity relates to the ability of the algorithm to identify positive results. In fact, it is the probability of detecting CAD, assuming that the patient actually has the disease. Specificity, on the other hand, relates to the ability of the algorithm to detect negative results. In other words, it is the probability of a negative algorithm output, given that the sample is healthy. Accuracy is the overall portion of correctly identified samples.

### 4.3. Evaluation

The impact of different features on disease presence is not uniform. This impact can be measured with the Gini index. The Gini index measures the inequality between the values of a distribution. Accordingly, higher values of the Gini index for a feature indicate its prevalence in causing the disease. Tables 4 -6 show the Gini index per feature, presenting the impact of the features on the stenosis of the LAD, LCX, and RCA, respectively. As Table 4 demonstrates, the most effective features on the LAD stenosis were region with regional wall motion abnormality (RWMA), ejection fraction (EF), age, valvular heart disease (VHD), erythrocyte sedimentation rate (ESR), lymph, neutrophils (Neut.), hypertension (HTN), potassium (K), white blood cells (WBC), and fasting blood sugar (FBS), respectively.

**Table 4.** Gini Index for LAD artery

Feature	Weight
**Region with RWMA^a^**	1
**EF^a^**	0.905
**Age**	0.628
**VHD^a^**	0.388
**ESR^a^**	0.357
**Lymph.^a^**	0.338
**Neut.^a^**	0.269
**HTN^a^**	0.246
**K^a^**	0.213
**WBC^a^**	0.176
**FBS^a^**	0.17
**CR^a^**	0.168
**TG^a^**	0.161
**BUN^a^**	0.159
**Na^a^**	0.154
**HDL^a^**	0.114
**LDL^a^**	0.113
**DM^a^**	0.111
**BMI^a^**	0.098
**Weight**	0.095
**HB^a^**	0.088
**PLT^a^**	0.07
**Sex**	0.068
**Length**	0.067
**Thyroid disease**	0.062
**Current Smoker**	0.052
**EX-Smoker**	0.046
**CRF^a^**	0.036
**Obesity**	0.026
**CHF^a^**	0.015
**CVA^a^**	0.015
**DLP^a^**	0.014

^a^ Abbreviations: RWMA, regional wall motion abnormality; EF, ejection fraction; VHD, Valvular Heart Disease; ESR, erythrocyte sedimentation rate; Lymph, lymphocyte; Neut, Neutrophil; HTN, hyper tension; K, potassium; WBC, white blood cell; FBS, fasting blood sugar; CR, Creatine; TG, triglyceride; BUN, blood urea nitrogen; Na, sodium; HDL, high-density lipoprotein; LDL, low-density lipoprotein; DM, diabetes mellitus; BMI, body mass index; HB, hemoglobin; PLT, platelet; CRF, chronic renal failure; CHF, congestive heart Failure; CVA, cerebrovascular accident; DLP, dyslipidemia

As Table 5 illustrates, the most effective features on the LCX stenosis were age, creatine (CR), FBS, platelets (PLT), lymphocytes (Lymph.), blood urea nitrogen (BUN), triglyceride (TG), HTN, EF, diabetes mellitus (DM), ESR, and high-density lipoprotein (HDL).

**Table 5.** Gini Index for LCX artery

Feature	Weight
**Ag**e	1
**CR^a^**	0.533
**FBS^a^**	0.458
**PLT^a^**	0.417
**Lymph.^a^**	0.391
**BUN^a^**	0.381
**TG^a^**	0.381
**HTN^a^**	0.329
**EF^a^**	0.293
**DM^a^**	0.268
**ESR^a^**	0.257
**HDL^a^**	0.238
**Region with RWMA^a^**	0.206
**K^a^**	0.191
**Na^a^**	0.177
**Length**	0.176
**HB^a^**	0.173
**WBC^a^**	0.171
**BMI^a^**	0.168
**Neut.^a^**	0.157
**EX-Smoker**	0.146
**VHD^a^**	0.14
**LDL^a^**	0.14
**Weight**	0.137
**Sex**	0.126
**Airway Disease**	0.101
**CRF^a^**	0.069
**Thyroid Disease**	0.067

^a^ Abbreviations: CR, Creatine; FBS, fasting blood sugar; PLT, platelet; Lymph, lymphocyte; BUN, blood urea nitrogen; TG, triglyceride; HTN, hyper tension; EF, ejection fraction; DM, diabetes mellitus; ESR, erythrocyte sedimentation rate; HDL, high-density lipoprotein; RWMA, regional wall motion abnormality; K, potassium; Na, sodium; HB, hemoglobin; WBC, white blood cell; BMI, body mass index; Neut, neutrophil; VHD, Valvular Heart Disease; LDL, low-density lipoprotein; CRF, chronic renal failure

As Table 6 reveals, the effect of DM, age, Lymph., ESR, Neut., FBS, WBC, length, HTN, EF, TG, hemoglobin (HB), sex, and CR on the RCA stenosis was more than the other features.

**Table 6.** Gini Index for RCA artery

Feature	Weight
**DM^a^**	1
**Age**	0.852
**Lymph.^a^**	0.78
**ESR^a^**	0.741
**Neut.^a^**	0.721
**FBS^a^**	0.499
**WBC^a^**	0.408
**Length**	0.301
**HTN^a^**	0.298
**EF^a^**	0.272
**TG^a^**	0.26
**HB^a^**	0.256
**Sex**	0.201
**CR^a^**	0.187
**PLTa**	0.175
**K^a^**	0.175
**VHD^a^**	0.168
**Weight**	0.166
**HDL^a^**	0.151
**Na^a^**	0.145
**BMI^a^**	0.135
**LDL^a^**	0.11
**Region with RWMA^a^**	0.078
**CHF^a^**	0.075
**BUN^a^**	0.075
**CVA^a^**	0.049

^a^ Abbreviations: DM, diabetes mellitus; Lymph, lymphocyte; ESR, erythrocyte sedimentation rate; Neut, neutrophil; FBS, fasting blood sugar; WBC, white blood cell; HTN, hyper tension; EF, ejection fraction; TG, triglyceride; HB, hemoglobin; CR, Creatine; PLT, platelet; K, potassium; VHD, Valvular Heart Disease; HDL, high-density lipoprotein; Na, sodium; BMI, body mass index; LDL, low-density lipoprotein; RWMA, regional wall motion abnormality; CHF, congestive heart failure; BUN, blood urea nitrogen; CVA, cerebrovascular accident

A comparison of the three tables reveals that some features such as age, EF, ESR, Lymph., and HTN affected all the arteries significantly. Also, regarding the results, diagnosing the stenosis of the LAD and RCA was easier owing to several high-impact features which affect them. A comparison of the performance measures of the algorithms for the diagnosis of the stenosis of the three arteries is portrayed in Tables 7 and 8.

**Table 7.** Performance of C4.5 for Diagnosis of Arteries Stenosis

Artery	Accuracy	Sensitivity	Specificity
**LAD**	77.83%	86.44%	65.87%
**LCX**	58.77%	72.28%	37.82%
**RCA**	68.31%	54.39%	76.72%

**Table 8.** Performance of Bagging for Diagnosis of Arteries Stenosis

Artery	Accuracy	Sensitivity	Specificity
**LAD**	78.51%	85.88%	68.25%
**LCX**	61.46%	78.26%	35.29%
**RCA**	68.96%	50%	80.42%

As Table 7 displays, the C4.5 algorithm diagnosed the stenosis of the LAD more accurately than the two other arteries. Furthermore, the diagnosis of the RCA stenosis was more accurate than that of the LCX. Sensitivity of the LCX and LAD was higher than their specificity, unlike the RCA. This means that for the two former arteries, the C4.5 offered a low false negative rate. As Table 8 shows, the accuracy of the Bagging algorithm for LAD stenosis diagnosis was higher than those of the two other arteries. In this algorithm, similar to the C4.5, sensitivity for the LAD and LCX was higher than specificity, unlike the RCA. The highest accuracy rates for the LAD, LCX, and RCA stenosis available in the literature belong to Babaoglu et al. ( 3 ), which are 73%, 64.85%, and 69.39%, respectively. As this table demonstrates, the accuracy for diagnosing the LAD stenosis was higher than those of the other similar studies and the accuracy for diagnosing the LCX and RCA stenosis was almost the same as those of the other similar studies. Even a small increase in accuracy can be beneficial, since the diagnosis of artery stenosis is extremely vital in the world of medicine (2).

In order to select the most important features, the Gini index and information gain were used. For this purpose, first, the features were sorted in two distinct groups based on these two metrics (Gini index and information gain). Thereafter, the 20 most important features based on each metric were selected. Finally, the C4.5 and Bagging algorithms were run on these two groups of selected features. The final results are illustrated in Tables 9 and 10.

**Table 9.** Performance of Bagging and C4.5 for Diagnosis of Artery Stenosis by Selecting Features Using Information Gain

Artery	Accuracy	Sensitivity	Specificity
**C4.5 Algorithm**			
**LAD**	76.56%	76.84%	76.19%
**LCX**	63.10%	70.11%	52.10%
**RCA**	63.68%	52.63%	70.37%
**Bagging Algorithm**			
**LAD**	79.54%	83.05%	74.60%
**LCX**	65.09%	73.37%	52.10%
**RCA**	66.31%	54.39%	73.54%

**Table 10.** Performance of Bagging and C4.5 for Diagnosis of Artery Stenosis by Selecting Features UsingGini Index

Artery	Accuracy	Sensitivity	Specificity
**C4.5 Algorithm**			
**LAD**	73.23%	76.84%	68.25%
**LCX**	60.43%	69.57%	46.22%
**RCA**	67.96%	53.51%	76.72%
**Bagging Algorithm**			
**LAD**	76.87%	81.36%	70.63%
**LCX**	63.11%	73.37%	47.06%
**RCA**	68.95%	56.14%	76.72%

A comparison of Tables 7, 8, 9 and 10 indicates that while feature selection decreased the accuracy of the LAD and RCA stenosis diagnosis, it had an opposite effect on the LCX. Furthermore, the use of features selected based on information gain enhanced the accuracy of the LAD stenosis diagnosis to 79.54%, which is higher than the figures reported by previous studies. Alizadehsani et al. ( 4 ) attained the accuracy rates of 74.20%, 63.76%, and 68.33% for the LAD, LCX, and RCA stenosis diagnoses, respectively, by using symptom and examination features as well as the ECG features.

The current study extracted some rules not only to evaluate dyslipidemia (DLP), TG, HB, and some echocardiographic features such as the EF but also to diagnose HTN and DM via the RapidMiner application. In these rules, which are shown below, S and C represent Support and Confidence, respectively. Support shows in what ratio of data the named features of a rule occur all together. The equality of Confidence to 1 shows that whenever the left side of a rule appears, the right side definitely occurs. In the following rules, males older than 45 years and females older than 55 years are considered Old since they are more prone to CAD occurrence. This categorization is based on Braunwald’s Heart Disease Book (2). HB is regarded as Low when it is lower than 14 for males and 12.5 for females, and high when it is higher than 17 for males and 15 for females (2).

## 5. Discussion

In the current study, the C4.5 and Bagging algorithms were used on laboratory and echocardiographic features on account of the fact that they are known as the best classification algorithms in data mining. Although laboratory and echocardiographic features play important roles in stenosis diagnosis (2), to the best of our knowledge, they have not been considered in any other previous works for the diagnosis of the stenosis of the LAD, LCX, and RCA, separately. Therefore, it was decided to assess the accuracy of these features to diagnose the stenosis of each of these three arteries separately. The results indicated that EF, age, Lymph and HTN were among the 10 most effective features on the stenosis of all of the arteries.

The accuracy rate obtained in the present study for the LAD stenosis diagnosis is higher than that of the most accurate method, proposed by Babaoglu et al. (3). Nonetheless, in LCX and RCA stenosis diagnoses, the results are quite the same. Finally, the current study succeeded in extracting some important rules to assess HB, HTN, TG, EF, DM, and DLP. To the best of our knowledge, the relationship between these features has not been taken into account in any other previous study. These studies include the ones conducted by Srinivas et al. (5), Ordonez et al. (6) and Sony et al. (7) who extracted some rules for the stenosis of each artery, separately. In the current study, the stenosis of the LAD, LCX, and RCA were examined separately. The C4.5 and Bagging algorithms were employed to analyze the dataset. To the best of our knowledge, this study presents the highest accuracy value for diagnosing the LAD stenosis in the available literature. The diagnostic accuracy may be further augmented by adding new features such as the ECG and examination features in future work.
